# Three new species of the genus *Neophyllomyza* Melander (Diptera, Milichiidae) from China, with a revised key to the Chinese species

**DOI:** 10.3897/zookeys.867.36247

**Published:** 2019-07-30

**Authors:** Yu-Qiang Xi, Ding Yang, Xin-Ming Yin

**Affiliations:** 1 Department of Entomology, Henan Agricultural University, No. 95 Wenhua Road, Jinshui District, Zhengzhou 450003, Henan Province, China Henan Agricultural University Zhengzhou China; 2 Department of Entomology, China Agricultural University, No. 2 Yuanmingyuan West Road, Haidian District, Beijing 100193, China China Agricultural University Beijing China

**Keywords:** Diptera, Milichiidae, *
Neophyllomyza
*, morphology, taxonomy

## Abstract

Three new species of the genus *Neophyllomyza*, *N. clavipalpis***sp. nov.**, *N. motuoensis***sp. nov.**, and *N. obtusa***sp. nov.**, are described from China. A revised key to the six Chinese species of *Neophyllomyza* is also presented.

## Introduction

*Neophyllomyza* Melander, 1913 is a small genus in the subfamily Phyllomyzinae with 13 known species, which are distributed the worldwide. There are three Palearctic species (Villeneuve 1920; Hendel 1924; Iwasa 2019), three Oriental species (Xi and Yang 2014), two Afrotropical species (Lamb 1914; Séguy 1938), two Australian species (Hendel 1907; Curran 1936), two Nearctic species (Melander 1913; Brochu and Wheeler 2009), and one Neotropical species (Williston 1896). Adults of *Neophyllomyza* are small acalyptrate flies. *Neophyllomyza* species are kleptoparasities, with adults mostly sucking at the prey of spiders or insects (Robinson and Robinson 1977; Sivinski and Stowe 1980).

Three species have been recorded from China (Xi and Yang 2014), *N. luteipalpis*, *N. lii*, and *N. tibetensis*. In this study, all known Chinese species are reviewed, and three new species are described.

## Materials and methods

Genitalia preparations were made by removing and macerating the apical portion of the abdomen in glacial acetic acid, then rinsing them in distilled water before storing them in glycerine filled microvials. After examination, genitalia were transferred to fresh glycerine and stored in a microvial on the pin below the specimen or moved to an ethanol tube together with the wet specimens. Specimens examined were deposited in the Entomological Museum of Henan Agricultural University (**HAU**), Zhengzhou; the Entomological Museum of China Agricultural University (**CAU**), Beijing. The general terminology follows McAlpine (1981) and Brake (2000). The following abbreviations are used:

**asc** apical scutellar seta(e),

**pa** postalar seta(e),

**bsc** basal scutellar seta(e),

**pos** postsutural seta(e),

**dc** dorsocentral seta(e),

**prs** presutural seta(e),

**h** humeral seta(e),

**prsc** prescutellar seta(e),

**ia** intraalar seta(e),

**sa** supraalar seta(e),

**kepsts** katepisternal seta(e),

**S** sternite,

**npl** notopleural seta(e),

**T** tergite.

## Taxonomy

### Neophyllomyza

Taxon classificationAnimaliaDipteraMilichiidae

Melander, 1913: 243.

d6db127c-3714-5841-b553-a2654e5fde03

#### Type species.

*Neophyllomyza quadricornis* Melander, 1913

#### Diagnosis.

Body small, 1.0–1.6 mm, brownish to dark brown. Postocellar setae cruciate or converging; paired cruciate setae present along the middle of the front; fronto-orbital setae extending to anterior margin of frons, the upper ones diverging, the lower converging; face excavated, cheeks narrow; palpus enlarged, compressed, apical setulae usually present; lunule small, bare.

### Neophyllomyza clavipalpis sp. nov.

Taxon classificationAnimaliaDipteraMilichiidae

f71c4d1f-1163-56e5-b751-3496c15ae869

http://zoobank.org/78E8652E-9140-4927-9FA4-1A2D9E6BFB10

Figures 1–6

#### Diagnosis.

**Figures 1–6.**
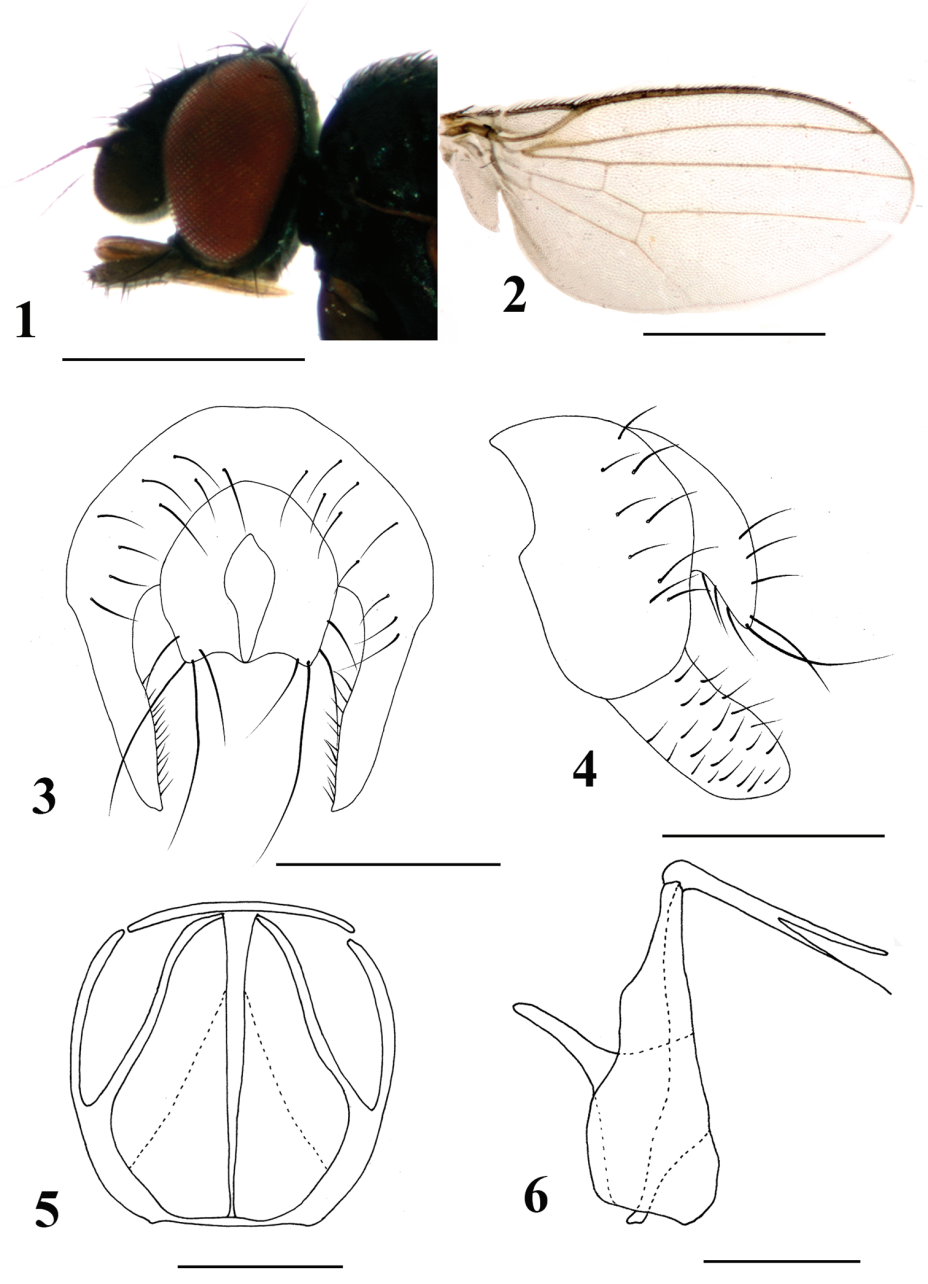
*Neophyllomyza clavipalpis* sp. nov. (male). **1** Head, lateral view **2** wing **3** epandrium, cerci, and surstyli, posterior view **4** epandrium, cerci, and surstyli, lateral view **5** genitalia, posterior view (hypandrium; phallapodemic; subepandrial) **6** genitalia in lateral view (hypandrium; phallapodemic; subepandrial). Scale bars: 0.5 mm (**1, 2**); 0.1 mm (**3–6**).

Gena relatively narrowed, approximately one-twelfth eye height; palpus brown, apically with black sparse setae (Fig. 1). Epandrium irregularly horseshoe-shaped with strong setae; surstylus elongate and margin with dense setulae. Hypandrium narrowed; phallapodeme rod-like. (Fig. 5).

#### Description.

*Male*. Body length 1.4–1.5 mm; wing length 1.3–1.4 mm.

Head (Fig. 1) darkish black with greyish microtomentum; orbital plate satiny blackish brown without microtomentum, ocellar triangle darkish black without microtomentum; lunule small, blackish with black margin. Posterior eye margin ventrally diverging from head margin; eye 1.4 times as high as long, gena approximately one-twelfth of eye height. Setae and setulae on head black; ocellar triangle with two ocellar setae and three short setae; frons with two orbital and two frontal setae, orbital setae lateroclinate and frontal setae medioclinate, three interfrontal setae; postocellar setae converging. Vibrissal angle relatively blunt; vibrissa strong, located above the level of lower margin of eye. Antenna darkish black, with microtomentum; pedicel with short black setae at middle and margin, setulae at margin longer than others, longest one approximately four times longer than others; first flagellomere with pubescence, approximately quadrate, apical margin smooth; arista two times as long as first flagellomere, black, pubescent very short. Proboscis geniculate, brownish-yellow, margin without setulae. Palpus rod-like, with blunt apex, narrow, approximately 0.2 mm, four times longer than wide; brown with short dense brownish pubescence, margin with black sparse setae.

Thorax black with grey microtomentum, except scutum shiny blackish-brown with sparse black microtomentum; scutellum darkish brown with grey microtomentum. Setae and setulae on thorax black; one h, two dc, one prsc, two npl, one prs, two pa, one kepsts (setulae at forward position); scutellum 1.5 times wider than long, with pair of asc and bsc, asc 2.5 times longer than bsc. Legs slender, darkish brown. Setae and setulae on legs black. Mid tibia with one black preapical dorsal seta. Wing hyaline (Fig. 2), unspotted; veins brown; Sc strong; M_1_ between r-m and dm-cu a little longer than dm-cu. Calypter yellowish, with dense brownish microtrichiae, margin with brownish setulae. Knob of halter brownish, stalk brown.

Abdomen darkish brown with grey microtomentum. Setae and setulae on abdomen black; TII-TV with setae, marginal setae slightly longer than others; sternites with sparse black setulae. SII generally luniform, SIII and SIV broad with middle narrower, SIV wider than SIII; SV very shallowly falciform, apical margin extends to basal margin. Male genitalia (Figs 3–6): epandrium irregularly falciform with strong setae; surstylus elongate and margin with dense setulae; hypandrium narrowed, ribbon pattern; phallapodeme pear-shaped; subepandrial sclerite well developed; cercus wide with long setae.

*Female*. Unknown.

#### Type material.

Holotype: ♂, **China**: Jilin, Antu, Changbai Mountain (42°27'14.20"N, 128°08'15.98"E; 1100 m), 26.VIII.2015, Yu-Qiang Xi (HAU). Paratype: 1 ♂, same data as holotype (HAU).

#### Distribution.

China (Jilin).

#### Etymology.

The specific name refers to the shape of the palpus.

#### Remarks.

This new species is somewhat similar to *N. acyglossa* (Villeneuve), but can be differentiated from the latter by the following features: M_1_ between r-m and dm-cu 1.8 times as long as dm-cu; knob of halter brownish, stalk brown; epandrium irregularly luniform. In *N. acyglossa*, M_1_ between r-m and dm-cu is 1.2 times as long as dm-cu; knob of halter dark brown, stalk blackish brown; epandrium very finely crescentic (Villeneuve 1920).

### Neophyllomyza motuoensis sp. nov.

Taxon classificationAnimaliaDipteraMilichiidae

c7ac0384-bbfb-53be-be95-3cf2cec60a3c

http://zoobank.org/19C2D14C-E401-4577-90CD-A5E107C771AA

Figures 7–12

#### Diagnosis.

**Figures 7–12.**
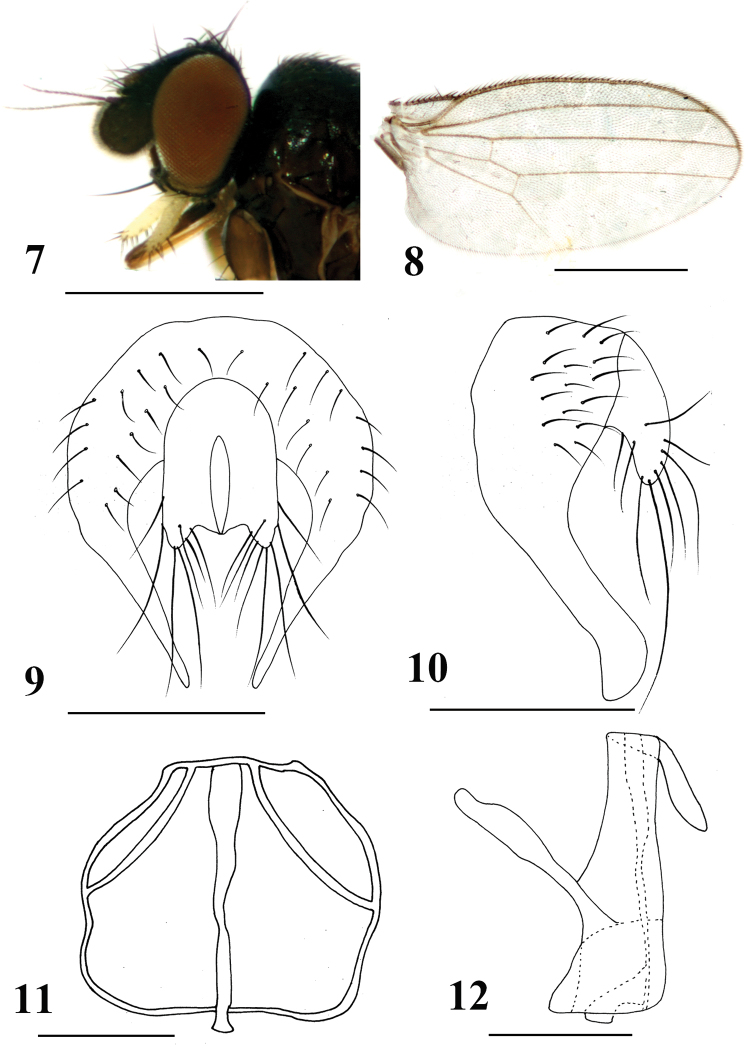
*Neophyllomyza motuoensis* sp. nov. (male). **7** Head, lateral view **8** wing **9** epandrium, cerci, and surstyli, posterior view **10** epandrium, cerci, and surstyli, lateral view **11** genitalia, posterior view (hypandrium; phallapodemic; subepandrial) **12** genitalia, lateral view (hypandrium; phallapodemic; subepandrial). Scale bars: 0.5 mm (**7, 8**); 0.1 mm (**9–12**).

Gena relatively narrowed, approximately one-ninth of eye height; palpus yellowish with short dense brownish pubescence (Fig. 7). Surstylus elongate, apically slightly blunted; hypandrium narrowed, irregularly quadrate; subepandrial sclerite well-developed. Cercus enlarged with long setae (Figs 9–12).

#### Description.

*Male*. Body length 1.2–1.5 mm; wing length 1.3–1.6 mm.

Head (Fig. 7) blackish-brown with greyish microtomentum; orbital plate subshiny blackish-brown without microtomentum; ocellar triangle darkish brown without microtomentum; lunule small, darkish yellow with black margin. Posterior eye margin ventrally diverging from head margin; eye 1.5 times as high as long, gena approximately one-ninth of eye height. Setae and setulae on head black; ocellar triangle with two ocellar setae and three short setae; frons with two orbital and two frontal setae, orbital setae lateroclinate and frontal setae medioclinate, three interfrontal setae; postocellar setae converging. Vibrissal angle relatively blunt; vibrissa strong, located at the level of lower margin of eye. Antenna darkish brown with microtomentum; pedicel with short black setae at middle and margin, setulae at margin longer than others, longest one approximately 2.5 times longer than others; first flagellomere with pubescence, approximately quadrate; arista 2.5 times as long as first flagellomere, darkish brown, distinctly short pubescence. Proboscis geniculate, darkish brown, margin without setulae. Palpus oblong with blunt apex in lateral view, approximately 0.2 mm, 4 times longer than wide; yellowish with short dense brownish pubescence, apically with short and long sparse black setae.

Thorax darkish brown with grey microtomentum, except scutum shiny blackish-brown with sparse black microtomentum; scutellum darkish brown with grey microtomentum. Setae and setulae on thorax black; one h, two dc, one prsc, two npl, one prs, one sa, one pa, one kepsts (a row of setulae at forward position); scutellum 1.5 times wider than long, with pair of asc and bsc, asc 2.5 times longer than bsc. Legs slender, darkish brown except tarsi yellow. Setae and setulae on legs black. Mid tibia with one black preapical dorsal seta. Wing hyaline (Fig. 8); veins brown; Sc strong; M_1_ between r-m and dm-cu longer than dm-cu. Calypter yellowish, with dense brownish microtrichae, margin with brownish setulae. Knob of halter yellowish white, stalk brownish.

Abdomen darkish brown with grey microtomentum. Setae and setulae on abdomen black; TII-T V with setae, rowed marginal setae slightly longer than others; sternites with sparse setulae. SII generally luniform, apically slightly blunt; SIII oblong; SIV irregularly quadrate; SV falciform, apical margin extends to basal margin extremely at mid. Male genitalia (Figs 9–12): epandrium irregularly semi-circular, with strong setae; surstylus elongate, apically slightly blunted, margin without dense setulae; hypandrium narrowed, irregularly quadrate; subepandrial sclerite well-developed; phallapodeme linearly pear-shaped. Cercus enlarged with long setae.

*Female*. Body length 1.3–1.6 mm; wing length 1.4–1.6 mm. Similar to male. Female terminalia: TVIII distinctly narrowed, blackish with dense short microtomentum. Supra-anal plate irregularly quadrate; subanal plate luniform, apically wide and blunt, with sparse setulae. Cercus long, apically nearly circular; black, with sparse setulae.

#### Type material.

Holotype: ♂, **China**: Tibet, Motuo, 80 K (29°39'20.75"N, 95°29'17.59"E, 1000 m), 23.VII.2012, Xuan-Kun Li (CAU). Paratypes: 1 ♂, 1 ♀, same data as holotype (CAU); 1 ♂, 2 ♀♀, China, Tibet, Motuo, Beibeng (29°14'30.75"N, 95°10'18.59"E, 700 m), 30.VII.2012, Wen-Liang Li (CAU).

#### Distribution.

China (Tibet).

#### Etymology.

The specific name refers to the type locality of Motuo County.

#### Remarks.

This new species is somewhat similar to *N. lii* Xi et Yang, 2014 but can be differentiated from the latter by the following features: postocellar setae converging; three interfrontal setae; scutellum 1.5 times wider than long, with pair of asc and bsc, asc 2.5 times longer than bsc. In *N. lii*, postocellar setae cruciate; four interfrontal setae; scutellum two times wider than long, with pair of asc and bsc, asc three times longer than bsc (Xi and Yang 2014).

### Neophyllomyza obtusa sp. nov.

Taxon classificationAnimaliaDipteraMilichiidae

1daa7a8d-0b51-55ff-8a8c-2b586af9bc8b

http://zoobank.org/1ABDE481-E006-43CD-BADB-BBA0722E53C6

Figures 13–18

#### Diagnosis.

**Figures 13–18.**
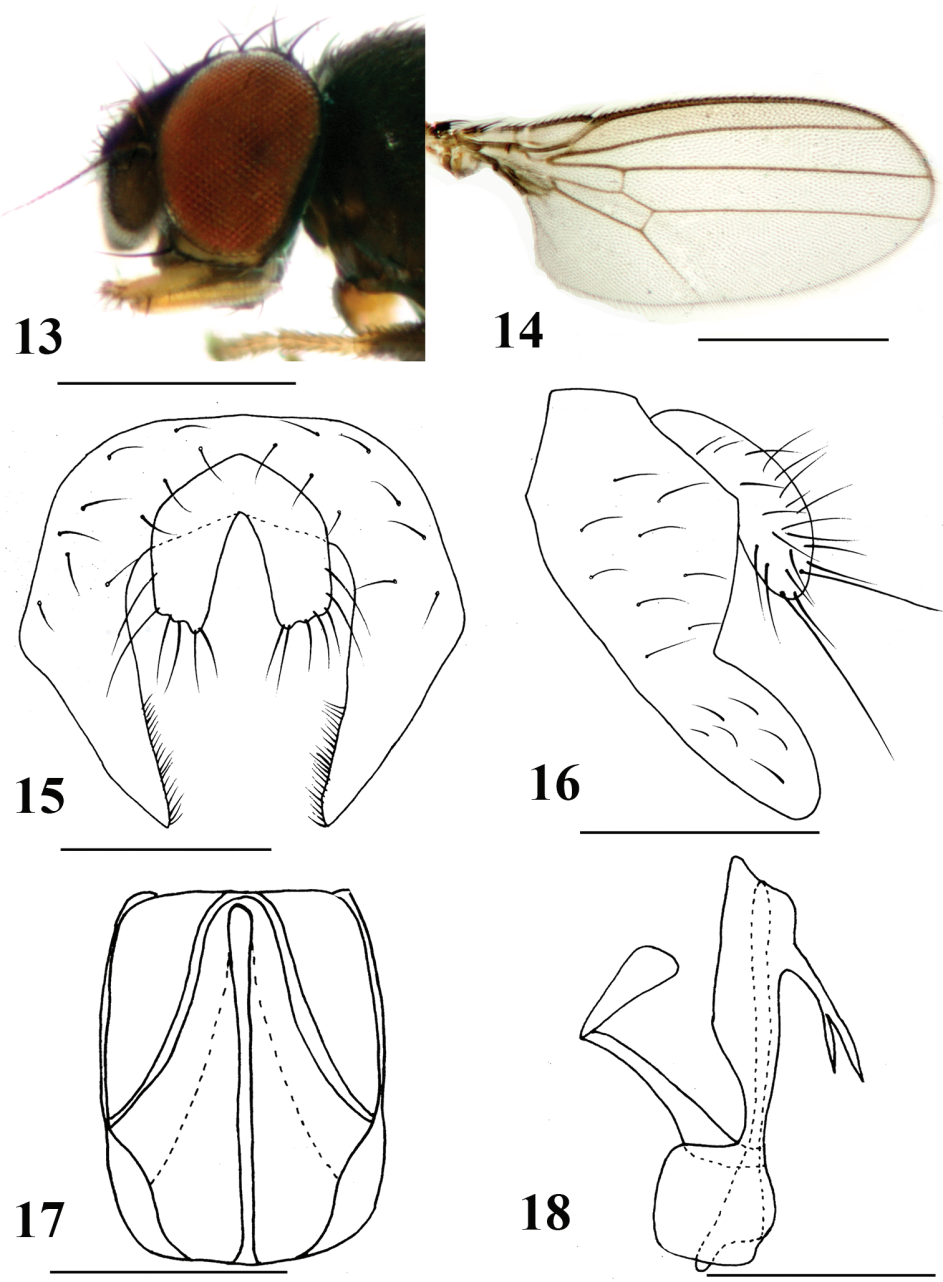
*Neophyllomyza obtusa* sp. nov. (male). **13** Head, lateral view **14** wing **15** epandrium, cerci, and surstyli, posterior view **16** epandrium, cerci, and surstyli, lateral view **17** genitalia, posterior view (hypandrium; phallapodemic; subepandrial) **18** genitalia, lateral view (hypandrium; phallapodemic; subepandrial). Scale bars: 0.5 mm (**13, 14**); 0.1 mm (**15–18**).

Gena narrowed, approximately one-fifteenth eye height; palpus darkish yellow and terminal with short and long sparse black setae (Fig. 13). Surstylus wide, apically blunt; hypandrium narrowed, finely luniform; subepandrial sclerite well-developed, irregularly quadrate. Cercus enlarged with long setae (Figs 15–18).

#### Description.

*Male*. Body length 1.1–1.2 mm; wing length 1.1–1.2 mm.

Head (Fig. 13) black with greyish microtomentum; orbital plate subshiny black without microtomentum; ocellar triangle black without microtomentum; lunule small, yellow with brown margin. Posterior eye margin ventrally diverging from head margin; eye 1.4 times as high as long, gena approximately one-fifteenth eye height. Setae and setulae on head black; ocellar triangle with two ocellar setae and three short setae; frons with two orbital and two frontal setae, orbital setae lateroclinate and frontal setae medioclinate, three interfrontal setae; postocellar setae cruciate. Vibrissal angle blunt; vibrissa strong, located at level of lower margin of eye. Antenna brown with microtomentum; pedicel with short black setulae at middle and margin, setulae at margin longer than others, longest one approximately 3.5 times longer than others; first flagellomere with pubescence, irregularly circular; arista four times as long as first flagellomere, brown, distinctly pubescent. Proboscis long, geniculate, darkish yellow, margin with short sparse black setulae. Palpus oblong with blunt apex in lateral view, less than 0.2 mm, four times longer than wide; darkish yellow, with short dense brownish pubescence, apically with short and long sparse black setae.

Thorax darkish brown with grey microtomentum, except mesonotum shiny darkish brown with sparse black microtomentum; scutellum blackish-brown with grey microtomentum. Setae and setulae on thorax black; one h, two dc, one prsc, two npl, one prs, one pos, one ia, one sa, one pa, one kepsts (setulae at forward position); scutellum 1.6 times wider than long, with pair of asc and bsc, asc three times longer than bsc. Legs slender, darkish brown, except tarsi darkish yellow. Setae and setulae on legs black. Mid tibia with 1 black preapical dorsal seta. Wing hyaline (Fig. 14); veins brown; Sc strong; M_1_ between r-m and dm-cu longer than dm-cu. Calypter yellowish with dense brownish microtrichae, margin with brownish setulae. Knob of halter brown, stalk brownish.

Abdomen darkish brown with grey microtomentum. Setae and setulae on abdomen black; TII-T V with setae at posterior 3/4, marginal setae longer than others; sternites with sparse setulae. SII generally luniform, apical margin slightly blunt; SIII oblong; SIV very broadly broad with middle narrower, apical margin slightly wider than basal margin; SV broadly broad with middle narrower, the apical margin smooth and depressed falciform. Male genitalia (Figs 15–18): epandrium irregularly luniform, with strong setae; surstylus wide, apically blunt; hypandrium narrowed, very shallowly falciform; subepandrial sclerite well-developed, irregularly quadrate; phallapodemic sclerite linearly irregularly quadrato-rhombiform. Cercus enlarged with long setae.

*Female*. Unknown.

#### Type material.

Holotype: ♂, **China**: Guangxi, Fangchenggang, Shangsi (21°54'27.25"N, 107°54'18.81"E, 1400 m), 17.V.2013, Xing-Yue Liu (CAU). Paratypes: 2 ♂♂, same data as holotype (CAU).

#### Distribution.

China (Guangxi).

#### Etymology.

The specific name refers to the shape of the vibrissal angle.

#### Remarks.

This new species is somewhat similar to *N. leanderi* (Hendel, 1924), but can be differentiated from the latter by the following features: knob of halter brown, stalk brownish; surstylus wide, apically blunt; cercus wide. In *N. leanderi*, knob of halter yellowish, stalk yellow; surstylus relatively narrow and long; cercus long and thin (Hendel 1924).

### Neophyllomyza luteipalpis

Taxon classificationAnimaliaDipteraMilichiidae

Xi & Yang, 2014

d1cc7dc3-2477-558f-a297-3c2acc55870f

Figures 19–24

Neophyllomyza luteipalpis Xi et Yang, 2014: 1641. Type-locality: China (Yunnan).

#### Diagnosis.

Body, 1.4–1.6 mm; wing, 1.4–1.6 mm. Gena very narrowed, approximately one-nineteenth eye height. Palpus darkish yellow, apically brownish and with strong setae. Epandrium irregularly hemispherical with strong setae; surstylus elongate and margin with dense setulae; cercus bifurcated and with long setae.

**Figures 19–24.**
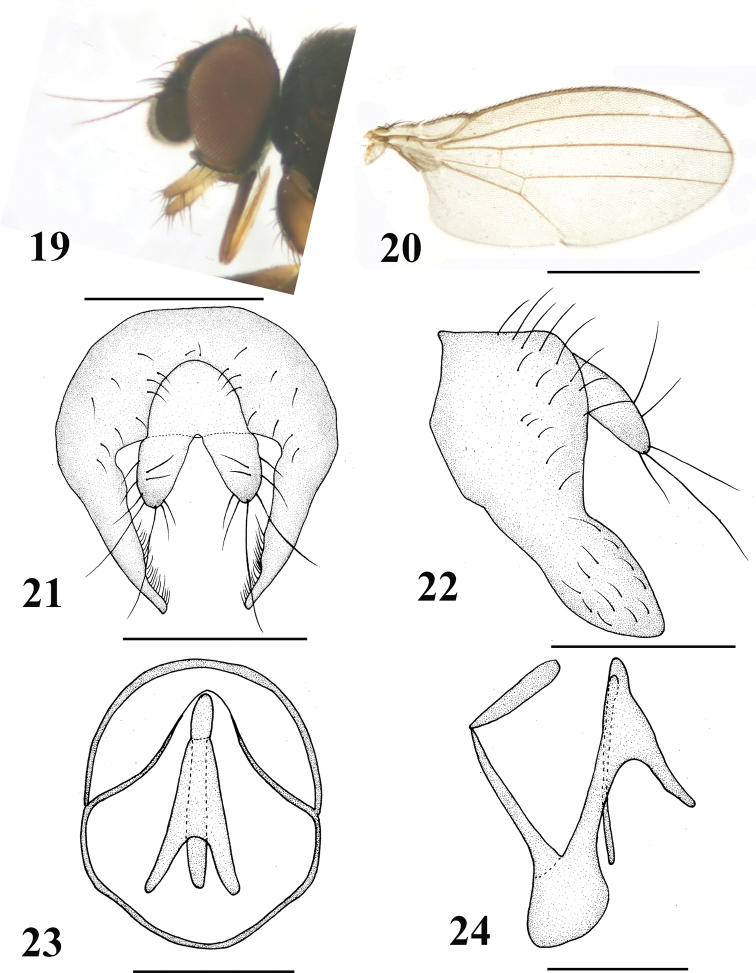
*Neophyllomyza luteipalpis* Xi & Yang, 2014 (male). **19** Head, lateral view **20** wing **21** epandrium, cerci, and surstyli, posterior view **22** epandrium, cerci, and surstyli, lateral view **23** genitalia, posterior view (hypandrium; phallapodemic; subepandrial) **24** genitalia, lateral view (hypandrium; phallapodemic; subepandrial). Scale bars: 0.5 mm (**19, 20**); 0.1 mm (**21–24**).

#### Material.

1 ♂, **China**: Yunnan, Baoshan, Baihualing (25°17'32.85"N, 98°48'23.08"E; 1575 m), 12.VII.2012, Wen-Liang Li (CAU); 1 ♂, **China**: Yunnan, Yingjiang, Xima (24°36'56.63"N, 97°39'23.09"E; 1390 m), 5.V.2012, Wen-Liang Li (CAU); 1 ♂, **China**: Yunnan, Tengchong, Guangming (25°29'15.92"N, 98°32'28.86"E; 1835 m), 7.V.2012, Wen-Liang Li (CAU); 3 ♀♀, **China**: Yunnan, Baoshan, Baihualing (25°17'32.85"N, 98°48'23.08"E; 1575 m), 16.V.2012, Wen-Liang Li (CAU).

#### Distribution.

China (Yunnan).

### Neophyllomyza lii

Taxon classificationAnimaliaDipteraMilichiidae

Xi & Yang, 2014

7d95924e-6e22-50e0-b40d-99860a141032

Figures 25–30

Neophyllomyza lii Xi et Yang, 2014: 1643. Type-locality: China (Yunnan).

#### Diagnosis.

Body, 1.4–1.6 mm; wing, 1.2–1.4 mm. Gena narrowed, approximately one-twelfth eye height; palpus brownish; apically with both short and long sparse black setae; epandrium irregularly hemispherical, with strong setae; surstylus elongate with apex slightly acute, margin with dense setulae; cercus bifurcated and elongate, with long setae.

**Figures 25–30.**
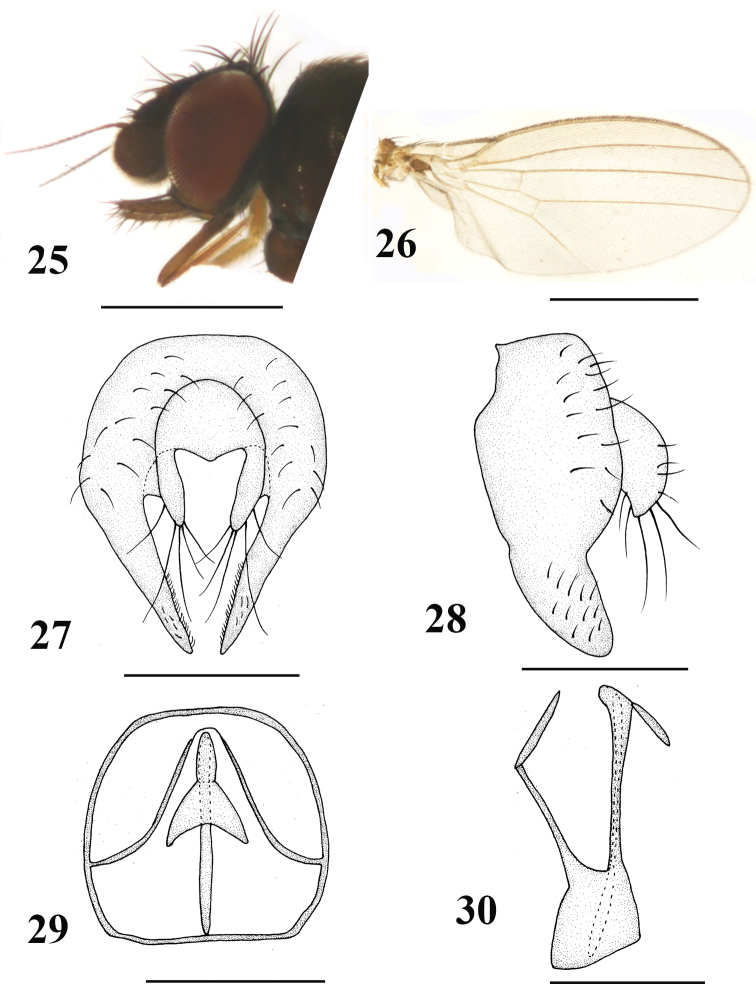
*Neophyllomyza lii* Xi & Yang, 2014 (male). **25** Head, lateral view **26** wing **27** epandrium, cerci, and surstyli, posterior view **28** epandrium, cerci, and surstyli, lateral view **29** genitalia, posterior view (hypandrium; phallapodemic; subepandrial) **30** genitalia, lateral view (hypandrium; phallapodemic; subepandrial). Scale bars: 0.5 mm (**25, 26**); 0.1 mm (**27–30**).

#### Material.

1 ♂, **China**: Yunnan, Yingjing, Tongbiguan (24°36'56.63"N 97°39'23.09"E, 1340 m), 1.V.2012, Wen-Liang Li (CAU); 1 ♂, **China**: Yunnan, Baoshan, Dahaoping (25°19'18.84"N, 100°08'32.04"E, 1925 m), 11.V.2012, Wen-Liang Li (CAU).

#### Distribution.

China (Yunnan).

### Neophyllomyza tibetensis

Taxon classificationAnimaliaDipteraMilichiidae

Xi & Yang, 2014

e7b49340-f574-52ea-a7a4-1bc176932778

Figures 31–36

Neophyllomyza tibetensis Xi et Yang, 2014: 1646. Type-locality: China (Tibet).

#### Diagnosis.

Body, 1.2–1.4 mm; wing, 1.2–1.4 mm. Gena relatively narrowed, approximately one-eighth eye height. Palpus darkish brown and apically with sparse black setae. Epandrium irregularly falciform, with strong setae; surstylus elongate and slightly blunted apically, margin with dense setulae; cercus broad with long setae.

**Figures 31–36.**
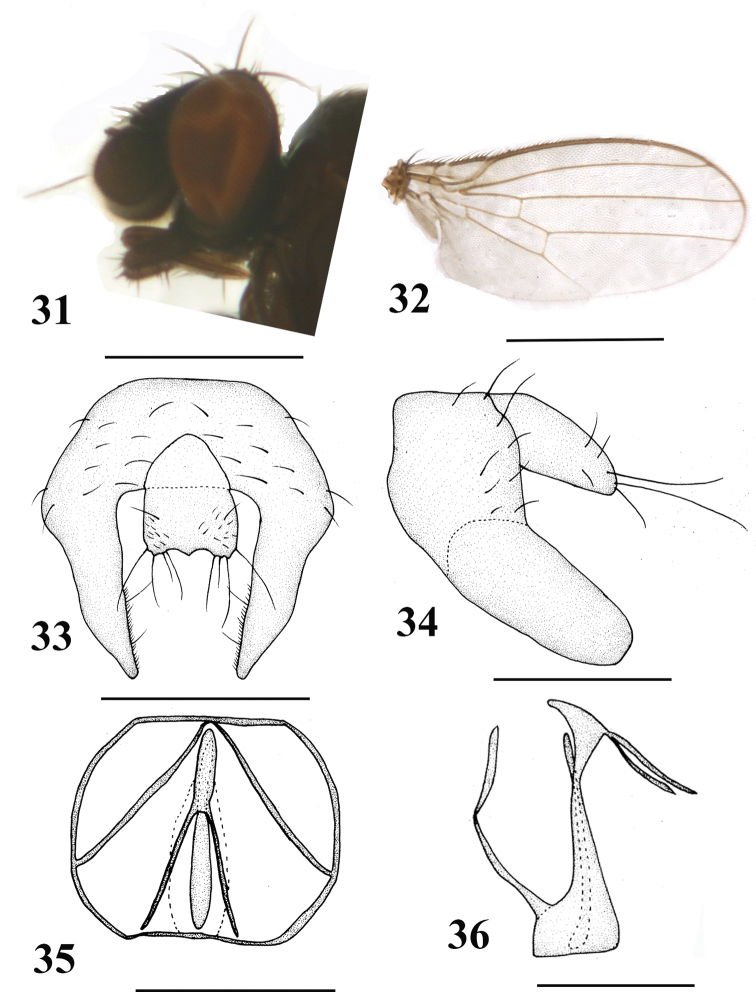
*Neophyllomyza tibetensis* Xi & Yang, 2014 (male). **31** Head, lateral view **32** wing **33** epandrium, cerci, and surstyli, posterior view **34** epandrium, cerci, and surstyli, lateral view **35** genitalia, posterior view (hypandrium; phallapodemic; subepandrial) **36** genitalia, lateral view (hypandrium; phallapodemic; subepandrial). Scale bars: 0.5 mm (**31, 32**); 0.1 mm (**33–36**).

#### Material.

1 ♂, **China**: Tibet, Linzhi, Bayi Town (29°38'32.58"N 94°21'32.55"E, 825 m), 5. V. 2012, Wen-Liang Li (CAU); 1 ♀, **China**: Tibet, Linzhi, Bayi Town (29°38'32.58"N, 94°21'32.55"E, 810 m), 30. VI. 2012, Xuan-Kun Li (CAU).

#### Distribution.

China (Tibet).

### Key to Chinese species (males) of *Neophyllomyza*

**Table:** 

1	Postocellar setae cruciate	**2**
–	Postocellar setae converging	**4**
2	Four interfrontal setae; eye 1.6 times as high as long	***N. luteipalpis* Xi & Yang**
–	Three interfrontal setae; eye 1.4 times as high as long	**3**
3	M_1_ between r-m and dm-cu 1.8 times as long as dm-cu; gena approximately one-twelfth of eye height	***N. lii* Xi & Yang**
–	M_1_ between r-m and dm-cu 1.5 times as long as dm-cu; gena approximately one-fifteenth of eye height	***N. obtusa* sp. nov.**
4	Arista 2 times as long as first flagellomere; vibrissa strong, located above the level of lower eye margin	***N. clavipalpis* sp. nov.**
–	Arista 2.5 times as long as first flagellomere; vibrissa strong, located at the level of lower eye margin	**5**
5	Gena approximately one-ninth of eye height; M_1_ between r-m and dm-cu 1.8 times as long as dm-cu ***N. motuoensis* sp. nov.**
–	Gena approximately one-seventh of eye height; M_1_ between r-m and dm-cu 1.3 times as long as dm-cu	***N. tibetensis* Xi & Yang**

### Faunistic remarks

Chinese species of *Neophyllomyza* are mainly distributed in Tibet and Yunnan Province, and *N. obtusa* sp. nov. and *N. clavipalpis* sp. nov. occur in Guanxi and Jilin Provinces; all of these represent new provincial records for *Neophyllomyza* in China. There are five species distributed in the Palaearctic Region (Villeneuve 1920; Hendel 1924; Xi and Yang 2014; Iwasa 2019), and three species (*N. clavipalpis* sp. nov., *N. motuoensis* sp. nov., and *N. tibetensis* Xi et Yang) from China in the Palaearctic Region. Three Chinese species are distributed in the Oriental Region, *N. obtusa* sp. nov., *N. luteipalpis* Xi et Yang, and *N. lii* Xi et Yang (Xi and Yang 2014). With further research, more species of *Neophyllomyza* will be found, because the Chinses fauna of Milichiidae is extraordinarily rich.

## Supplementary Material

XML Treatment for Neophyllomyza

XML Treatment for Neophyllomyza clavipalpis

XML Treatment for Neophyllomyza motuoensis

XML Treatment for Neophyllomyza obtusa

XML Treatment for Neophyllomyza luteipalpis

XML Treatment for Neophyllomyza lii

XML Treatment for Neophyllomyza tibetensis
